# Towards Full Thickness Small Intestinal Models: Incorporation of Stromal Cells

**DOI:** 10.1007/s13770-023-00600-6

**Published:** 2023-12-19

**Authors:** Melis Asal, Mila Rep, Hetty J. Bontkes, Sandra J. van Vliet, Reina E. Mebius, Susan Gibbs

**Affiliations:** 1 Department of Molecular Cell Biology and Immunology, Amsterdam UMC, Vrije Universiteit, Amsterdam, The Netherlands; 2 Laboratory Medical Immunology, Amsterdam University Medical Center, Amsterdam, The Netherlands; 3 Cancer Center Amsterdam, Amsterdam Infection and Immunity Institute, Amsterdam, The Netherlands; 4 Department of Oral Cell Biology, Academic Centre for Dentistry Amsterdam (ACTA), University of Amsterdam and Vrije Universiteit, Amsterdam, The Netherlands

**Keywords:** Human small intestine, Lamina propria, Stromal cells, *In vitro* model

## Abstract

**Introduction:**

Since small intestine is one of the major barriers of the human body, there is a need to develop reliable in vitro human small intestinal models. These models should incorporate both the epithelial and lamina propria compartments and have similar barrier properties compared to that of the human tissue. These properties are essential for various applications, such as studying cell–cell interaction, intestinal diseases and testing permeability and metabolism of drugs and other compounds. The small intestinal lamina propria contains multiple stromal cell populations with several important functions, such as secretion of extracellular matrix proteins and soluble mediators. In addition, stromal cells influence the intestinal epithelial barrier, support the intestinal stem cell niche and interact with immune cells.

**Methods:**

In this review, we provide an extensive overview on the different types of lamina propria stromal cells found in small intestine and describe a combination of molecular markers that can be used to distinguish each different stromal cell type. We focus on studies that incorporated stromal cells into human representative small intestine models cultured on transwells.

**Results and Conclusion:**

These models display enhanced epithelial morphology, increased cell proliferation and human-like barrier properties, such as low transepithelial electrical resistance (TEER) and intermediate permeability, thus better mimicking the native human small intestine than models only consisting of an epithelium which generally show high TEER and low permeability.

## Introduction

Human small intestine is one of the major barriers of the human body and is composed of an epithelial layer and an underlying lamina propria. The epithelium is important for digestion and absorption of nutrients [1, 2]. This columnar epithelial monolayer also forms a tight barrier and represents the first line of defense against foreign substances [3]. The structural integrity of the barrier for homeostasis of the intestine and the maintenance of mucosal immunity is maintained via tight junctions connecting the cells to each other, which control the diffusion along the paracellular pathway [4]. The small intestinal lamina propria and epithelium actively interact and regulate each other’s development and proliferation *in vivo* and this interaction has been recognized as crucial for the establishment of mucosal integrity [3, 5].

Animal models are often favored in preclinical studies, but they pose issues related to ethical concerns. Additionally, species differences between animals and humans can lead to misleading outcomes, posing a major challenge for the use of animal models in research [6]. There is therefore a need to develop *in vitro* small intestinal models with barrier properties similar to the human tissue in order to predict the safety and efficacy of drugs.

Multiple studies have shown that adding lamina propria stromal cells to *in vitro* intestinal models made of epithelium leads to changes in cell morphology and barrier properties [7–13]. These studies show a reduction in transepithelial electrical resistance (TEER) and an increase in permeability, bringing the barrier properties closer to that of the native human small intestine. In this review we first summarize the cell types found within the lamina propria and then discuss the current state of the art and challenges of incorporating these cell types into organotypic intestine models.

## Small intestinal lamina propria

**Fig. 1:**
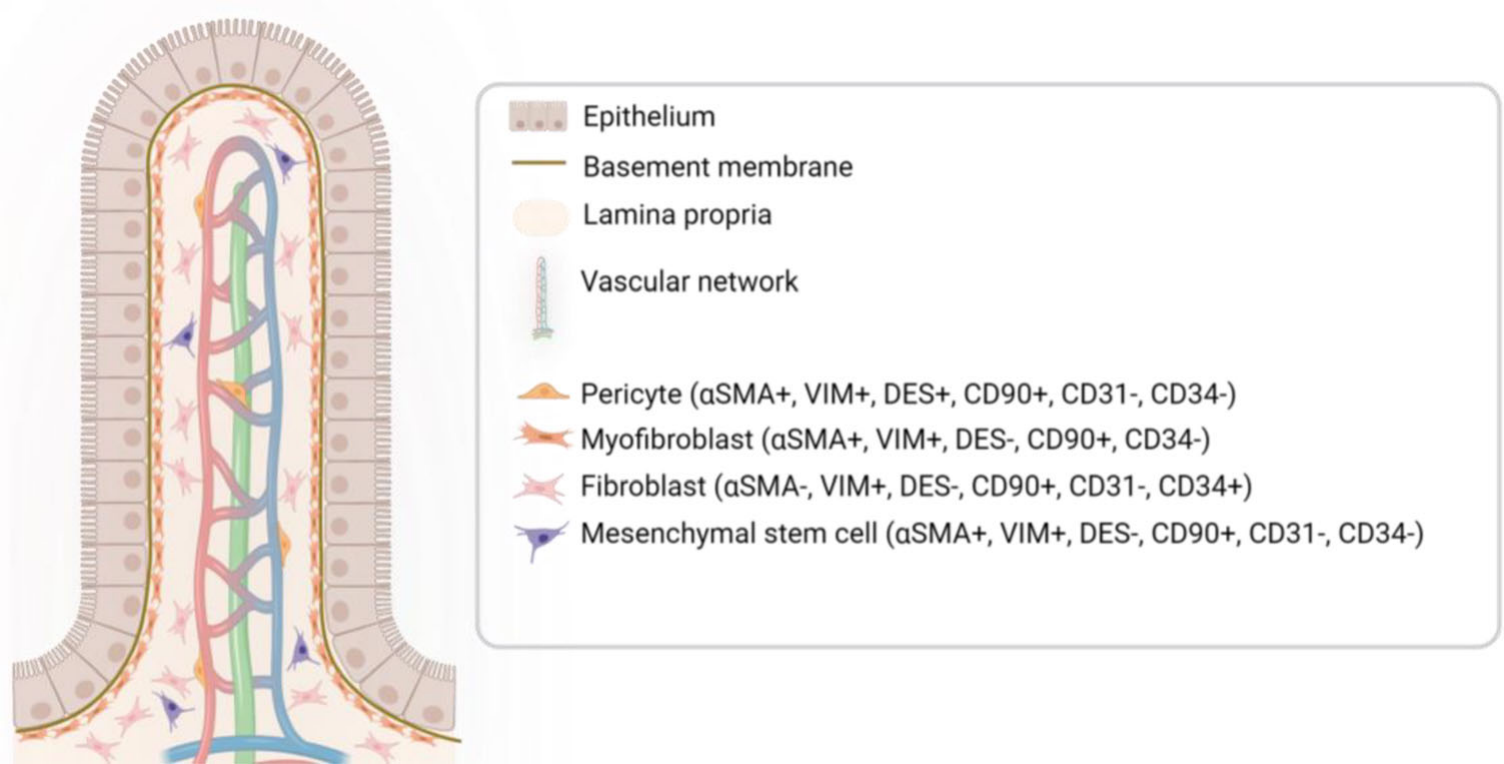
Architecture of the human small intestine. Intestinal epithelial cells are located above the basement membrane, which is positioned above the lamina propria, a highly vascular extracellular matrix containing multiple stromal cells with cell specific markers, namely fibroblasts, myofibroblasts, pericytes and mesenchymal stem cells. This figure was created in BioRender.com

Lamina propria of small intestine is the tissue that is located underneath the epithelium, with a basement membrane in between the two layers [11]. The acellular basement membrane dynamically regulates epithelial cell morphogenesis, cell differentiation and polarity, while being the structural base for villi, crypts and the microvasculature of the lamina propria [14, 15]. The basement membrane consists mainly of collagen type IV, laminin, nidogen and perlecan [16, 17]. The intestinal lamina propria is an extracellular matrix (ECM)-rich connective tissue hosting mesenchymal stem cells, fibroblasts, myofibroblasts, smooth muscle cells, pericytes, neurons and enteric glial cells [3, 18, 19]. It also hosts lymphatics and capillaries [12]. The ECM of the lamina propria is made up of collagen, elastin [20], glycoproteins, proteoglycans [11], glycosaminoglycans [21] and growth factors. Next to being a physical scaffold for cells, the lamina propria offers mechanical and chemical signals essential for cellular processes [22], and forms and supports the intestinal epithelial stem cell niche [23]. Moreover, the lamina propria provides support to the epithelium by secreting factors regulating epithelial proliferation and differentiation [24, 25]. A distinctive characteristic of this layer is that it contains a substantial number of immune cells, like T and B lymphocytes, innate lymphoid cells [26], plasma cells [27], mast cells [28], macrophages, dendritic cells [29], NK cells, granulocytes [3], eosinophils [20] and neutrophils [30]. Figure 1 shows the architecture of the human small intestinal mucosa layer and characteristics of the cells found within it.

## Characteristics of small intestinal lamina propria stromal cells

Intestinal stromal cells are mainly the fibroblasts, myofibroblasts, pericytes and stem cells, which are non-hematopoietic, non-epithelial, non-endothelial cells [18, 19, 31].

Fibroblasts in lamina propria are identified based on their morphology and various molecular markers. They are non-hematopoietic (CD45^−^), non-endothelial (CD31^−^) cells [32] that express the surface fibroblast marker CD90 (also called Thy-1), the intermediate filament protein vimentin and CD34 [33], but do not express alpha-smooth muscle actin (α-SMA) and the smooth muscle cell marker desmin [32, 34]. Fibroblasts within the lamina propria produce multiple ECM proteins [11, 35], including collagen type I, III, IV, fibronectin, hyaluronan [32], elastin-tropoelastin [36] and decorin [37], which are fundamental for the maintenance of the structural integrity of the intestine [33]. Furthermore, these cells fulfill crucial functions in the regulation of proliferation [24], migration, differentiation [38] and barrier properties of epithelial cells [7, 9, 12]. Moreover, they play a key role in healing and regeneration by secreting the growth factors GM-CSF and G-CSF [39], and various cytokines and chemokines [11, 35].

Myofibroblasts of the lamina propria are recognized as cells expressing CD90 [40], vimentin, α-SMA [41], smooth muscle heavy chain myosin (myosin-11) [42] and tenascin-C (TN-C) [43]. These cells have a smooth muscle appearance, but stain negative for the smooth muscle cell markers desmin, smoothelin and caldesmon [44]. Myofibroblasts have fundamental regulatory functions, such as regulating the epithelial cell development and remodeling and repairing impaired tissue after injury [25]. Contracting myofibroblasts account for the contraction of villi, leading to effective fluid transfer and cell shedding from the epithelium [15, 33, 45]. Myofibroblasts of the intestine produce the ECM proteins collagen type I and III, as well as the following growth factors; TGF-α, TGF-β, KGF, EGF, IGF [44], PDGF [33] and HGF [24]. To our knowledge the immunological role of the small intestinal myofibroblasts has yet to be investigated.

Pericytes are located around blood endothelial cells and can be identified by their expression of α-SMA, desmin, melanoma chondroitin sulfate proteoglycan (MCSP) and PDGF receptor beta (PDGFR-β). These cells maintain the growth and function of vascular and lymphatic endothelial cells, play roles in vascular contraction and support and produce PDGF [33, 46].

Bone-marrow derived mesenchymal stem cells (MSCs) are the common precursors of the mesenchymal lineage. They are classically defined as CD45^−^, CD34^−^ [47], α-SMA^+^ [48] and CD90^+^ cells [34, 49]. They are also plastic-adherent when maintained in standard culture conditions and express CD105 [50], CD73 [51] and vimentin, but not CD14, CD11b, CD79α, CD19 or HLA-DR surface molecules [52]. MSCs are important in the regulation of intestinal morphogenesis, management of epithelial proliferation and differentiation in the stem cell niche [34]. They can differentiate into fibroblasts [53], myofibroblasts [13] and pericytes [54]. Moreover, they are capable of producing the ECM proteins collagen type IV, laminins, nidogens and perlecan [35], and the growth factor R-spondin 3 [55]. Figure 1 summarizes the expression of each molecular marker by the various lamina propria stromal cells.

## Small intestinal lamina propria stromal cells and the immune system

Multiple studies suggest that the intestinal mucosa harbors significant immunomodulatory functions [3, 5, 19, 33]. As a location of exposure to both harmless and potentially threatening materials, the intestinal mucosa needs to integrate a combination of signals to balance immune responses [3]. It is known that intestinal stromal cells dynamically interact with immune cells.

In the small human intestine, a tolerogenic environment is achieved through various pathways of communication between the intestinal epithelial cells, the stromal cells and the immune cells. Small intestinal stromal cells can directly crosstalk with immune cells under steady-state and inflamed circumstances by producing various soluble mediators [33, 56–58]. The secretion of chemokines and cytokines within the lamina propria is important to create a gradient to support the migration and recruitment of a broad variety of immune cells, such as monocytes, macrophages, dendritic cells, basophils, eosinophils, neutrophils, NK cells, T and B cells [3]. This migration of immune cells is facilitated through the scaffold of ECM molecules produced by intestinal stromal cells [59]. An overview of the soluble mediators known to be secreted by small intestinal stromal cells can be found in Table 1. It is important to note that more secretory molecules were identified in studies focused on colon. Additional studies should be carried out to identify each cell type that secretes each molecule.

**Table 1:** Soluble mediators secreted by human small intestine lamina propria stromal cells under steady-state and inflamed conditions

Soluble mediators	Target cells	Steady-state	Inflamed
CCL2	Monocytes and basophils	[23]	[23, 64, 65]
CCL7	Monocytes and eosinophils	NS	[64]
CCL8	Monocytes, lymphocytes, basophils and eosinophils	NS	[64]
CCL11	Eosinophils	[66]	[64, 66]
CCL13	Monocytes, lymphocytes, basophils and eosinophils	NS	[64]
CCL19	T cells and B cells	[23, 66]	[23, 66]
CCL21	T cells	[66]	[66]
CXCL1	Neutrophils	[23, 67]	[23, 64, 67]
CXCL2	Neutrophils, basophils, eosinophils and hematopoietic stem cells	[23, 66]	[23, 64, 66]
CXCL3	Neutrophils	NS	[64]
CXCL5	Neutrophils	NS	[64]
CXCL6	Neutrophils	[67]	[64]
CXCL8	Neutrophils, basophils and T cells	NS	[64]
CXCL10	Monocytes, macrophages, T cells, NK cells and dendritic cells	NS	[66]
CXCL12	Monocytes and lymphocytes	[66]	[64, 66]
CXCL13	Monocytes, neutrophils and B cells	[23, 65, 66]	[23, 64–66]
CXCL14	Neutrophils and dendritic cells	NS	[64]
GM-CSF	Macrophages and dendritic cells	[61]	[64]
IL-6	B cells	[23, 65, 68]	[23, 64–66, 68, 69]
IL-7	T cells and B cells	NS	[23]
IL-11	T cells and B cells	[65]	[64, 65]
IL-34	Monocytes and macrophages	NS	[64]

This table is adapted from [3]

CCL chemokine ligands, CXCL chemokine (C-X-C motif) ligands, GM-CSF granulocyte macrophage colony-stimulating factor, IL interleukin, NS not studied

Some studies indicate the complex cell-to-cell interactions that may occur in the lamina propria between stromal and immune cell types regarding local tolerance [60]. This can be achieved by glycoprotein-38^+^CD31^−^ intestinal lamina propria stromal cells that stimulate CD103^+^ mucosal dendritic cells to make retinoic acid (RA) [61]. RA plays an important balancing role in supporting regulatory T cells while inhibiting Th17 differentiation in the intestinal mucosa [62, 63]. Interestingly, the stromal cells are also influenced by the dendritic cells, as their interaction stimulates the production of GM-CSF by stromal cells [61]. Colonic stromal cells play roles in mucosal immunity by expressing Toll-like receptors and by producing cytokines and chemokines. Furthermore, they act like non-professional antigen-presenting cells by expressing MHC class II [3, 5]. Whether small intestine stromal cells play a similar role in immune regulation is not yet clear as there are, to our knowledge, no existing studies in literature.

## *In vitro* small intestinal models

Intestine organotypic models often make use of a transwell culture system. These models involve culturing cells on a semipermeable membrane, which separates the apical (luminal) and basolateral compartments to mimic the intestinal barrier. The most commonly used *in vitro* intestinal model is an epithelial barrier model based on the human colon carcinoma derived epithelial cell line Caco-2. Caco-2 cells, when seeded on a transwell insert, can spontaneously differentiate into a monolayer of cells with a brush border resembling the intestinal absorptive enterocytes and form a barrier [64, 65]. This model is simple to make and provides consistent results [8]. Nonetheless, it has a major disadvantage. The Caco-2 monolayer model shows exceptionally high TEER values; TEER of Caco-2 monolayers is higher than 1000 Ω*cm^2^, while the TEER for human small intestinal tissue ranges only from 50 to 100 Ω*cm^2^. The human *in vivo* barrier is thus much more permeable compared to Caco-2 models [66].

To overcome this issue, different groups incorporated other cell types into their *in vitro* intestinal models. HT29-MTX cells, which imitate the small intestinal goblet cells, when incorporated lead to lower TEER values [7, 8, 12].

**Fig. 2:**
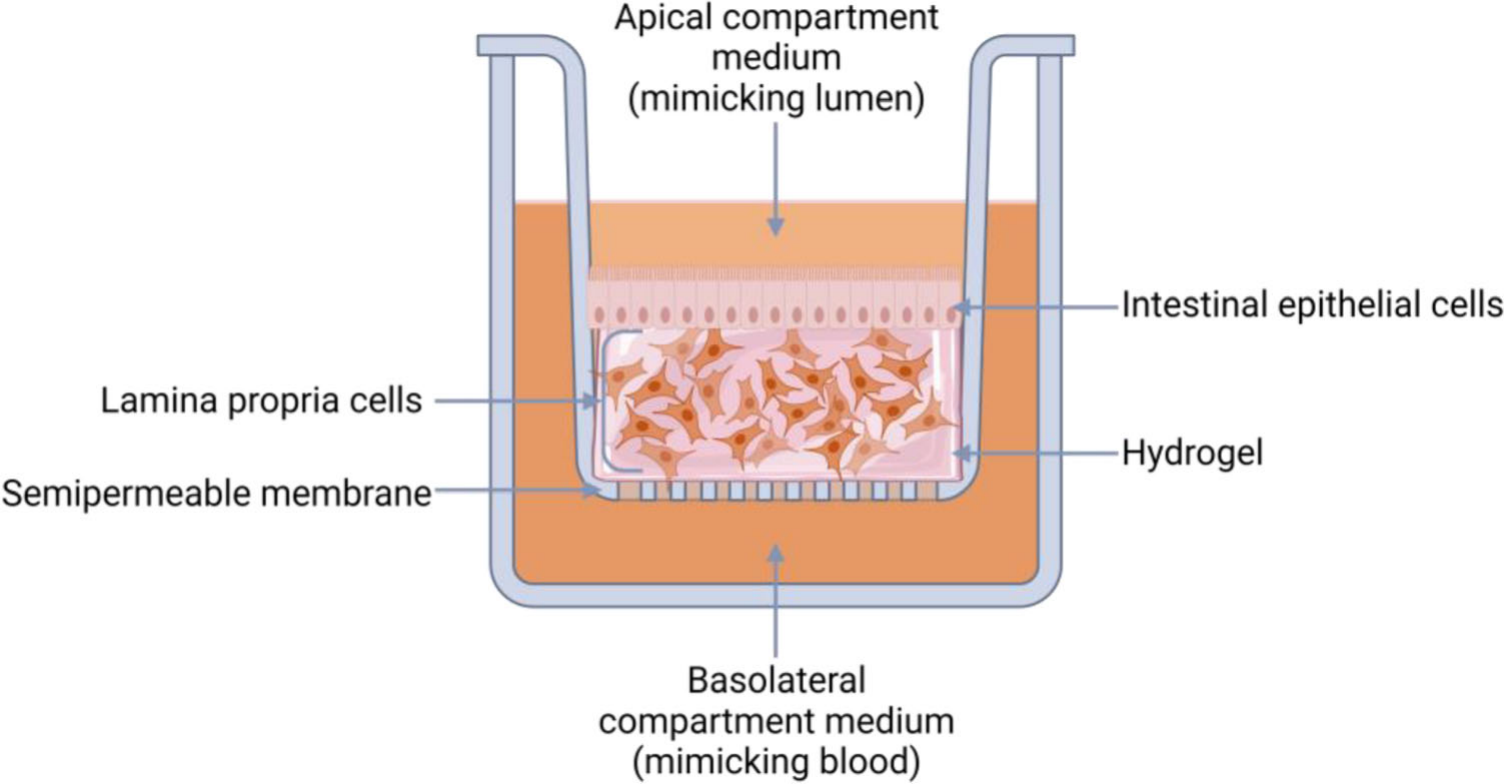
Schematic representation of the transwell based intestinal model incorporating the lamina propria. The figure illustrates a cross-section of the model. In the upper compartment, the intestinal epithelial barrier is depicted as a monolayer of polarized epithelial cells. Beneath the epithelial layer is the lamina propria layer consisting of a hydrogel populated with cells. This construct is placed on a semipermeable transwell membrane which separates the upper and lower compartments, allowing for custom designed culture media to be used for mimicking the lumen and blood microenvironments. This figure was created in BioRender.com

A major enhancement of the Caco-2 model has been achieved by adding a stromal layer underneath the Caco-2 cells, thereby permitting crosstalk between the two cell types [7, 11]. Of note, to our knowledge, all intestine models reported to date which incorporate the stromal layer make use of the *in vitro* transwell culture system (Table 2). Incorporation of intestinal stromal cells, such as fibroblasts or myofibroblasts, into such transwell based intestinal models have been shown to affect ECM production, cell morphology and, most importantly, barrier properties [7–13]. This approach involves adding within the transwell different lamina propria cell types (e.g., immune cells and/or stromal cells) into a hydrogel, which recapitulates the ECM, thus forming the basolateral side, and seeding intestinal epithelial cells (cell lines or organoid derived cells) onto the hydrogel, thus forming the apical side. In this way the intestinal microenvironment is reconstructed (Fig. 2). These studies show a reduction in TEER and an increase in permeability, bringing the barrier properties closer to that of the native human small intestine.

**Table 2:** Overview of the three dimensional intestinal *in vitro* models with epithelium and lamina propria compartments

Epithelial layer	Lamina Propria layer	Scaffold	Barrier competency			Reference
			Permeability	TEER	TEER compared to native small intestine	
Caco-2 and HT29-MTX cells	Human primary embryonic fibroblasts Human THP-1 derived macrophages	Rat tail collagen	^*^app. 11 × 10^–6^ cm/s c: app. 4.5 × 10^–6^ cm/s	app. 500 Ω*cm^2^ c: app. 2000 Ω*cm^2^	↑	[7]
Caco-2 and HT29-MTX cells	Human CCD-18co intestinal myofibroblasts	Matrigel	NS	app. 250 Ω*cm^2^ c: app. 1750 Ω*cm^2^	↑	[8]
Human primary ileum organoids	Human primary ileum fibroblasts	Collagen coated plates	NS	70–200 Ω*cm^2^ c: NS	→	[9]
Human primary intestinal organoids	Human CC-2902 intestinal myofibroblasts	Collagen coated plates	NS	NS	NS	[10]
Caco-2 cells	Human CCD-18co intestinal myofibroblasts or human neonatal dermal fibroblasts	Alvetex® scaffold	^+^app. 2.5 × 10^–6^ cm/s c: app. 0.5 × 10^–6^ cm/s	50–200 Ω*cm^2^ c: app. 2000 Ω*cm^2^	→	[11]
Caco-2 and HT29-MTX cells	Human primary intestinal fibroblasts	Rat tail collagen	^*^app. 2.4 × 10^–6^ cm/s c: < 0.5 × 10^–7^ cm/s	app. 200 Ω*cm^2^ c: app. 700 Ω*cm^2^	↑	[12]
Caco-2 cells	3T3 mouse embryonic fibroblasts	Rat tail collagen type 1	^+^1.84 ± 0.20 × 10^–7^ cm/s c: 6.47 ± 1.59 × 10^–8^ cm/s	app. 1000 Ω*cm^2^ c: app. 2200 Ω*cm^2^	↑	[13]

TEER of small intestine (*in vivo*): 50–100Ωcm^2^ [73]. Models of [9] and [10] were maintained at the air–liquid interface after 4 days submerged, the rest of the models were submerged. All models were cultured on porous transwell membranes

c Control, * Fluorescein, + Lucifer yellow ↑ Higher, → Similar, NS not studied

In addition to the enhanced barrier properties, several studies reported ECM protein expression such as collagen type I, III and IV, fibronectin and laminin by intestinal stromal cells [8–12]. Some studies confirmed activity of various transporters (ABCB1, ABCG2, ABCC1, ABCC2) which play critical roles in a wide range of physiological processes, including drug absorption, distribution and elimination [11]. One study explored two interaction mechanisms: paracrine signaling and direct contact in three dimensional (3D) co-culture. Using conditioned media from fibroblasts, paracrine signaling did not significantly affect epithelial morphology, as shown by transmission electron microscopy. However, notable changes in 3D co-culture included enhanced polarization, formation of basement membrane-like attachments and straightened lateral membranes resembling *in vivo* intestinal mucosa [11]. In another study, the presence of lamina propria cells in a hydrogel contributed to enhanced cell proliferation of epithelial cells in the 3D organotypic cultures with weaker tight junctions between the epithelial cells [8]. In addition to this, other studies noted proliferation of fibroblasts in the 3D organotypic model [12].

Although different absolute TEER values of the studied models were observed, all models showed lower TEER values than their control Caco-2 monolayer. Some studies also measured the permeability of lucifer yellow or fluorescein by the permeation coefficients (*P*_app_) in cm/s and found inversely correlating higher permeability values compared to their control consisting of a Caco-2 monolayer. These studies are summarized in Table 2. Creating and culturing transwell based organotypic models is accompanied by a number of challenges. These include the pros and cons on whether to use cell lines, which have the limitation in that they are of cancer origin and do not replicate the cellular diversity found in the intestine, versus the use of primary cells, which are accompanied with complex culture logistics and high donor variation. The choice of scaffold also needs to be considered with the simplest being a hydrogel and complexity increasing when porosity, villi and crypt architecture are included. Until now, intestine models which incorporate a lamina propria are reconstructed and grown in a transwell culture system. This model enables the microenvironment to be mimicked by using culture medium mimicking the lumen on the apical side and culture medium mimicking the microvascular nutrients on the basolateral side [67, 68]. Such models will be vital in the future to ensure the development of a functional and physiologically relevant intestinal barrier with proper tight junction formation and selective permeability, replicating the dynamic and multicellular environment and capturing the complex physiological cues of the native intestine, and predicting responses to stimuli or treatments.

## Discussion

Studies in recent decades have discovered the broad range of functions of the specific populations within the human small intestinal stromal cells [18, 19, 33, 57, 61]. Our review provides the combination of molecular markers that can be used to distinguish the different populations. There is no single molecular marker fully distinctive for one of the intestinal stromal cell types. This might be due to the high plasticity of mesenchymal cell types or the impact of the physiological context on marker expression.

Various intestinal models incorporating stromal cells have been described. All of these studies showed more native small intestine-like results, such as secretion of ECM components, enhanced epithelial cell proliferation, lower TEER and higher permeability, thus making them more representative of the human small intestine.

Incorporation of stromal cells into a matrix below the epithelial monolayer leads to the secretion of ECM components such as collagen I, III and IV, fibronectin and laminin. This is in line with the knowledge that stromal cells display an active role in the preservation of the overlying epithelial cells through the construction of their own ECM [11, 35].

Proliferation of epithelial cells might be due to the capacity of stromal cells to secrete multiple growth factors [24, 33, 44]. A similar process was identified in skin models, where keratinocyte proliferation was stimulated and epidermal differentiation was improved in the presence of fibroblasts. The same trend was apparent when keratinocytes were cultured in medium derived from dermal models or keratinocyte-fibroblast co-cultures, suggesting the release of soluble factors that stimulate proliferation from fibroblasts [69]. The underlying mechanisms regarding stromal cell-epithelial interactions have so far not been fully elucidated, but some efforts in this direction have been made. One study showed that a direct paracrine action of HGF and KGF released from human colonic fibroblasts or other stromal cells might account for the proliferative effect exerted by the co-cultured intestinal stromal cells [24]. The exact pathways and the factors contributing to the proliferation of small intestine epithelial cells still needs to be examined.

All of the reviewed intestinal *in vitro* models displayed lower TEER values, but to different degrees compared to the gold standard Caco-2 monolayers. The differences can be explained by the influence of the scaffold and equipment used to measure TEER. Synthetic or natural scaffolds, as well as decellularized matrices, have been shown to give various noteworthy signals to cells and affect their *in vitro* behavior [70–72]. Besides, there are other factors affecting TEER of Caco-2 monolayers, including medium components, culture time, passage of the cells and temperature [67, 68]. In addition to this, the reviewed models incorporated only fibroblasts or myofibroblasts, while small intestine is a complex structure with various cell types. Lack of the cell complexity might lead to discrepancies in the models. In line with the decrease in TEER, all studies indicated an increased permeability of fluorescein or lucifer yellow. This is in line with the lower TEER values, indicating decreased tight junction integrity of the epithelial layer. Overall, TEER values of the reviewed models integrating lamina propria cells were closer to human small intestine *in vivo* than the Caco-2 monolayer model, indicating that intestinal epithelial integrity is greatly influenced by the stromal cells.

A number of the described models use colon cells or cells of animal origin, which greatly hinders the representativeness of the developed human small intestine models, as the TEER and permeability of native colon is different from that of native small intestine and there are known interspecies differences. Moreover, Caco-2 and HT29-MTX cells have a cancer origin and are known to harbor genetic mutations [73]. In addition to this, using a single cell type of epithelial or lamina propria cells to mimic the small intestine does not adequately represent the complex interplay between the different cell types [30]. Since primary human intestinal organoids can differentiate into all epithelial cell types, using organoid derived cells would make the models more representative of the native small intestine. Out of all of the mentioned intestinal models, only two used primary organoids. These studies used an air–liquid interface to culture the cells, which is an interesting approach as this is not physiological for small intestine. Under air–liquid interface culture conditions, cells formed structures resembling villi according to the authors. However, these structures contain multilayers of epithelial cells, as observed in air-exposed skin cultures [74]. In native intestine, on the other hand, the epithelium forms a strict monolayer and is not air-exposed.

Future challenges would include incorporating microbiome, peristaltic dynamic flow and crypt-villus architecture. Such current models only incorporating the epithelium are starting to be developed [75–78]. However, none yet has been described to include the lamina propria with living cells.

In summary, this review highlights the importance of incorporating stromal cells into small intestine models to achieve more *in vivo*-like epithelial barrier features. In the future, these models should be further improved by incorporating all stromal cell types (fibroblasts, myofibroblasts, pericytes and mesenchymal stem cells) into the stromal compartments to better mimic the complexity of the human small intestine. In addition to stromal cells, immune cells, which are highly abundant in the small intestinal lamina propria, should be incorporated to render the models immune competent.

## Data Availability

The datasets generated during and/or analysed during the current study are available from the corresponding author on reasonable request.

## References

[1] Hornbuckle WE, Simpson KW, Tennant BC (2008). Gastrointestinal function: clinical biochemistry of domestic animals.

[2] Balimane PV, Chong S (2005). Cell culture-based models for intestinal permeability: a critique. Drug Discov Today.

[3] Pasztoi M, Ohnmacht C (2022). Tissue niches formed by intestinal mesenchymal stromal cells in mucosal homeostasis and immunity. Int J Mol Sci.

[4] Yu Q-H, Yang Q (2009). Diversity of tight junctions (TJs) between gastrointestinal epithelial cells and their function in maintaining the mucosal barrier. Cell Biol Int.

[5] Owens B, Simmons A (2013). Intestinal stromal cells in mucosal immunity and homeostasis. Mucosal Immunol.

[6] Hoffmann JC, Pawlowski NN, Kühl AA, Höhne W, Zeitz M (2002). Animal models of inflammatory bowel disease: an overview. Pathobiology.

[7] Li N, Wang D, Sui Z, Qi X, Ji L, Wang X (2013). Development of an improved three-dimensional in vitro intestinal mucosa model for drug absorption evaluation. Tissue Eng Part C Methods.

[8] Pereira C, Araújo F, Barrias CC, Granja PL, Sarmento B (2015). Dissecting stromal-epithelial interactions in a 3D in vitro cellularized intestinal model for permeability studies. Biomaterials.

[9] Ayehunie S, Landry T, Stevens Z, Armento A, Hayden P, Klausner M (2018). Human primary cell-based organotypic microtissues for modeling small intestinal drug absorption. Pharm Res.

[10] Peters MF, Landry T, Pin C, Maratea K, Dick C, Wagoner MP (2019). Human 3D gastrointestinal microtissue barrier function as a predictor of drug-induced diarrhea. Toxicol Sci.

[11] Darling NJ, Mobbs CL, González-Hau AL, Freer M, Przyborski S (2020). Bioengineering novel in vitro co-culture models that represent the human intestinal mucosa with improved Caco-2 structure and barrier function. Front Bioeng Biotechnol.

[12] Macedo MH, Martínez E, Barrias CC, Sarmento B (2020). Development of an improved 3D in vitro intestinal model to perform permeability studies of paracellular compounds. Front Bioeng Biotechnol.

[13] Zhang J, Penny J, Lu JR (2020). Development of a novel in vitro 3D intestinal model for permeability evaluations. Int J Food Sci Nutr.

[14] Hynes RO (2009). The extracellular matrix: not just pretty fibrils. Science.

[15] Ensari A, Marsh MN (2018). Exploring the villus. GHFBB.

[16] Miner JH, Li C, Mudd JL, Go G, Sutherland AE (2004). Compositional and structural requirements for laminin and basement membranes during mouse embryo implantation and gastrulation. Development.

[17] Pöschl E, Schlötzer-Schrehardt U, Brachvogel B, Saito K, Ninomiya Y, Mayer U (2004). Collagen IV is essential for basement membrane stability but dispensable for initiation of its assembly during early development. Development.

[18] Powell D, Pinchuk I, Saada J, Chen X, Mifflin R (2011). Mesenchymal cells of the intestinal lamina propria. Annu Rev Physiol.

[19] Roulis M, Flavell RA (2016). Fibroblasts and myofibroblasts of the intestinal lamina propria in physiology and disease. Differentiation.

[20] Boudry G, Yang P-C, Perdue MH, Johnson L (2004). Small intestine. Anatomy: encyclopedia of gastroenterology.

[21] Pender S, Lionetti P, Murch S, Wathan N, MacDonald T (1996). Proteolytic degradation of intestinal mucosal extracellular matrix after lamina propria T cell activation. Gut.

[22] Kim Y, Ko H, Kwon IK, Shin K (2016). Extracellular matrix revisited: roles in tissue engineering. Int Neurourol J.

[23] Stzepourginski I, Nigro G, Jacob J-M, Dulauroy S, Sansonetti PJ, Eberl G (2017). CD34^+^ mesenchymal cells are a major component of the intestinal stem cells niche at homeostasis and after injury. Proc Natl Acad Sci.

[24] Göke M, Kanai M, Podolsky DK (1998). Intestinal fibroblasts regulate intestinal epithelial cell proliferation via hepatocyte growth factor. Am J Physiol Gastrointest.

[25] Desmoulière A, Chaponnier C, Gabbiani G (2005). Tissue repair, contraction, and the myofibroblast. Wound Repair Regen.

[26] Fan H, Wang A, Wang Y, Sun Y, Han J, Chen W (2019). Innate lymphoid cells: regulators of gut barrier function and immune homeostasis. J Immunol Res.

[27] Dunn-Walters DK, Boursier L, Spencer J (1997). Hypermutation, diversity and dissemination of human intestinal lamina propria plasma cells. Eur J Immunol.

[28] Befus AD, Dyck N, Goodacre R, Bienenstock J (1987). Mast cells from the human intestinal lamina propria. Isolation, histochemical subtypes, and functional characterization. J Immunol.

[29] Vallon-Eberhard A, Landsman L, Yogev N, Verrier B, Jung S (2006). Transepithelial pathogen uptake into the small intestinal lamina propria. J Immunol.

[30] Gelberg HB (2014). Comparative anatomy, physiology, and mechanisms of disease production of the esophagus, stomach, and small intestine. Toxicol Pathol.

[31] Kinchen J, Chen HH, Parikh K, Antanaviciute A, Jagielowicz M, Fawkner-Corbett D (2018). Structural remodeling of the human colonic mesenchyme in inflammatory bowel disease. Cell.

[32] Pilling D, Fan T, Huang D, Kaul B, Gomer RH (2009). Identification of markers that distinguish monocyte-derived fibrocytes from monocytes, macrophages, and fibroblasts. PLoS ONE.

[33] Thomson CA, Nibbs RJ, McCoy KD, Mowat AM (2020). Immunological roles of intestinal mesenchymal cells. Immunology.

[34] Pinchuk I, Mifflin R, Saada J, Powell D (2010). Intestinal mesenchymal cells. Curr Gastroenterol Rep.

[35] Pompili S, Latella G, Gaudio E, Sferra R, Vetuschi A (2021). The charming world of the extracellular matrix: a dynamic and protective network of the intestinal wall. Front Med.

[36] Karsdal M (2019). Biochemistry of collagens, laminins and elastin: structure, function and biomarkers.

[37] Zhang W, Ge Y, Cheng Q, Zhang Q, Fang L, Zheng J (2018). Decorin is a pivotal effector in the extracellular matrix and tumour microenvironment. Oncotarget.

[38] Dignass AU, Tsunekawa S, Podolsky DK (1994). Fibroblast growth factors modulate intestinal epithelial cell growth and migration. Gastroenterology.

[39] Fibbe WE, Van Damme J, Billiau A, Duinkerken N, Lurvink E, Ralph P (1988). Human fibroblasts produce granulocyte-CSF, macrophage-CSF, and granulocyte-macrophage-CSF following stimulation by interleukin-1 and poly (rI). poly (rC). Blood.

[40] Saalbach A, Kraft R, Herrmann K, Haustein U-F, Anderegg U (1998). The monoclonal antibody AS02 recognizes a protein on human fibroblasts being highly homologous to Thy-1. Arch Dermatol Res.

[41] Andoh A, Bamba S, Brittan M, Fujiyama Y, Wright NA (2007). Role of intestinal subepithelial myofibroblasts in inflammation and regenerative response in the gut. Pharmacol Ther.

[42] Eyden B (2008). The myofibroblast: phenotypic characterization as a prerequisite to understanding its functions in translational medicine. J Cell Mol Med.

[43] De Wever O, Nguyen QD, Van Hoorde L, Bracke M, Bruyneel E, Gespach C (2004). Tenascin-C and SF/HGF produced by myofibroblasts in vitro provide convergent proinvasive signals to human colon cancer cells through RhoA and Rac. FASEB J.

[44] Powell D, Adegboyega P, Di Mari J, Mifflin R (2005). Epithelial cells and their neighbors I. Role of intestinal myofibroblasts in development, repair, and cancer. Am J Physiol Gastrointest.

[45] Powell D, Mifflin R, Valentich J, Crowe S, Saada J, West A (1999). Myofibroblasts. II. Intestinal subepithelial myofibroblasts. Am J Physiol Cell Physiol.

[46] Mifflin RC, Pinchuk IV, Saada JI, Powell DW (2011). Intestinal myofibroblasts: targets for stem cell therapy. Am J Physiol Gastrointest.

[47] Uccelli A, Moretta L, Pistoia V (2008). Mesenchymal stem cells in health and disease. Nat Rev Immunol.

[48] Bellini A, Mattoli S (2007). The role of the fibrocyte, a bone marrow-derived mesenchymal progenitor, in reactive and reparative fibroses. Lab Invest.

[49] Pham H, Tonai R, Wu M, Birtolo C, Chen M (2018). CD73, CD90, CD105 and Cadherin-11 RT-PCR screening for mesenchymal stem cells from cryopreserved human cord tissue. Int J Stem Cells.

[50] Barry FP, Boynton RE, Haynesworth S, Murphy JM, Zaia J (1999). The monoclonal antibody SH-2, raised against human mesenchymal stem cells, recognizes an epitope on endoglin (CD105). BBRC.

[51] Barry F, Boynton R, Murphy M, Zaia J (2001). The SH-3 and SH-4 antibodies recognize distinct epitopes on CD73 from human mesenchymal stem cells. BBRC.

[52] Gao Q, Wang L, Wang S, Huang B, Jing Y, Su J (2022). Bone marrow mesenchymal stromal cells: identification, classification, and differentiation. Front Cell Dev Biol.

[53] Lee CH, Moioli EK, Mao JJ. Fibroblastic differentiation of human mesenchymal stem cells using connective tissue growth factor. In: 2006 international conference of the IEEE engineering in medicine and biology Society 2006, pp. 775–778. 10.1109/IEMBS.2006.259866 PMC4035038 17946857

[54] Rajantie I, Ilmonen M, Alminaite A, Ozerdem U, Alitalo K, Salven P (2004). Adult bone marrow-derived cells recruited during angiogenesis comprise precursors for periendothelial vascular mural cells. Blood.

[55] Ogasawara R, Hashimoto D, Kimura S, Hayase E, Ara T, Takahashi S (2018). Intestinal lymphatic endothelial cells produce R-Spondin3. Sci Rep.

[56] Fritsch C, Simon-Assmann P, Kedinger M, Evans GS (1997). Cytokines modulate fibroblast phenotype and epithelial-stroma interactions in rat intestine. Gastroenterology.

[57] Nowarski R, Jackson R, Flavell RA (2017). The stromal intervention: regulation of immunity and inflammation at the epithelial-mesenchymal barrier. Cell.

[58] Koliaraki V, Prados A, Armaka M, Kollias G (2020). The mesenchymal context in inflammation, immunity and cancer. Nat Immunol.

[59] Vaday GG, Lider O (2000). Extracellular matrix moieties, cytokines, and enzymes: dynamic effects on immune cell behavior and inflammation. J Leukoc Biol.

[60] Roulis M, Kaklamanos A, Schernthanner M, Bielecki P, Zhao J, Kaffe E (2020). Paracrine orchestration of intestinal tumorigenesis by a mesenchymal niche. Nature.

[61] Vicente-Suarez I, Larange A, Reardon C, Matho M, Feau S, Chodaczek G (2015). Unique lamina propria stromal cells imprint the functional phenotype of mucosal dendritic cells. Mucosal Immunol.

[62] Mucida D, Park Y, Kim G, Turovskaya O, Scott I, Kronenberg M (2007). Reciprocal TH17 and regulatory T cell differentiation mediated by retinoic acid. Science.

[63] Coombes JL, Siddiqui KR, Arancibia-Cárcamo CV, Hall J, Sun C-M, Belkaid Y (2007). A functionally specialized population of mucosal CD103^+^ DCs induces Foxp3^+^ regulatory T cells via a TGF-β–and retinoic acid–dependent mechanism. J Exp Med.

[64] Basson MD, Turowski G, Emenaker NJ (1996). Regulation of human (Caco-2) intestinal epithelial cell differentiation by extracellular matrix proteins. Exp Cell Res.

[65] Peterson MD, Mooseker MS (1992). Characterization of the enterocyte-like brush border cytoskeleton of the C2_BBe_ clones of the human intestinal cell line, Caco-2. J Cell Sci.

[66] Béduneau A, Tempesta C, Fimbel S, Pellequer Y, Jannin V, Demarne F (2014). A tunable Caco-2/HT29-MTX co-culture model mimicking variable permeabilities of the human intestine obtained by an original seeding procedure. Eur J Pharm Biopharm.

[67] Sambuy Y, De Angelis I, Ranaldi G, Scarino M, Stammati A, Zucco F (2005). The Caco-2 cell line as a model of the intestinal barrier: influence of cell and culture-related factors on Caco-2 cell functional characteristics. Cell Biol Toxicol.

[68] Srinivasan B, Kolli AR, Esch MB, Abaci HE, Shuler ML, Hickman JJ (2015). TEER measurement techniques for in vitro barrier model systems. SLAS Technol.

[69] El-Ghalbzouri A, Gibbs S, Lamme E, Van Blitterswijk C, Ponec M (2002). Effect of fibroblasts on epidermal regeneration. Br J Dermatol.

[70] Sung JH, Yu J, Luo D, Shuler ML, March JC (2011). Microscale 3-D hydrogel scaffold for biomimetic gastrointestinal (GI) tract model. Lab Chip.

[71] Yu J, Carrier RL, March JC, Griffith LG (2014). Three dimensional human small intestine models for ADME-Tox studies. Drug Discov Today.

[72] Castaño AG, García-Díaz M, Torras N, Altay G, Comelles J, Martínez E (2019). Dynamic photopolymerization produces complex microstructures on hydrogels in a moldless approach to generate a 3D intestinal tissue model. Biofabrication.

[73] Ahmed D, Eide P, Eilertsen I, Danielsen S, Eknaes M, Hektoen M (2013). Epigenetic and genetic features of 24 colon cancer cell lines. Oncogenesis.

[74] Waaijman T, Breetveld M, Ulrich M, Middelkoop E, Scheper RJ, Gibbs S (2010). Use of a collagen–elastin matrix as transport carrier system to transfer proliferating epidermal cells to human dermis in vitro. Cell Transpl.

[75] Noel G, Baetz NW, Staab JF, Donowitz M, Kovbasnjuk O, Pasetti MF (2017). A primary human macrophage-enteroid co-culture model to investigate mucosal gut physiology and host-Pathogen Interactions. Sci Rep.

[76] Parlesak A, Haller D, Brinz S, Baeuerlein A, Bode C (2004). Modulation of cytokine release by differentiated CACO-2 cells in a compartmentalized coculture model with mononuclear leucocytes and nonpathogenic bacteria. Scand J Immunol.

[77] Wang Y, Gunasekara DB, Reed MI, DiSalvo M, Bultman SJ, Sims CE (2017). A microengineered collagen scaffold for generating a polarized crypt-villus architecture of human small intestinal epithelium. Biomater.

[78] Hinman SS, Wang Y, Kim R, Allbritton NL (2020). In vitro generation of self-renewing human intestinal epithelia over planar and shaped collagen hydrogels. Nat Protoc.

